# Medication Confidence and Emotional Distress in Older Adults with Type 2 Diabetes: A Moderated Mediation Approach

**DOI:** 10.1093/geroni/igaf122.3218

**Published:** 2025-12-31

**Authors:** Qiwei Li, Elias Mpofu, Xiaoli Li, Cheng Yin, Garikayi Chemhaka, Amos Mareverwa, Kaye Brock

**Affiliations:** California State University, Fresno, Fresno, California, United States; University of North Texas, Denton, Texas, United States; Southern Illinois University, Carbondale, Illinois, United States; University of North Texas, Denton, Texas, United States; University of North Texas, Denton, Texas, United States; University of North Texas, Denton, Texas, United States; The University of Sydney, Sydney, New South Wales, Australia

## Abstract

**Background and Aim:**

Emotional distress is common among individuals with type 2 diabetes (T2D) and would be higher with lower education levels and poor health literacy. High distress is linked to poor self-care and treatment adherence, increasing risks related to fatigue and comorbidities. Confidence in medication adherence and fatigue self-management would relief emotion distress with T2D, but evidence remains unclear. This study examines the relationship between medication confidence and emotional distress management, with fatigue management and comorbidities as mediators and education level as a moderator.

**Methods:**

Using a cross-sectional design, we analyzed data from the Diabetes Self-Management Program in Arlington, Texas. The study included 144 older adults (≥60 years; 30% male) with self-reported T2D. Confidence in emotional distress management, medication adherence, fatigue management, comorbidities, and education level were assessed. Multiple linear regression, parallel mediation, and moderated mediation analyses were conducted, controlling age, gender, and race.

**Results:**

Medication confidence was significantly associated with emotional distress management (β = 0.71, p < 0.01). Fatigue management confidence strongly mediated this relationship (β = 0.74, p < 0.01, R² = 0.54), while multimorbidity had no significant mediation effect. Education level moderated the direct effect, with stronger associations in participants with at least a bachelor’s degree. The overall model explained 67% of variance in emotional distress confidence (R² = 0.67).

**Conclusion and Implications:**

Interventions for emotional distress in older adults with T2D should enhance medication and fatigue management confidence. Education plays a key moderating role, underscoring the importance of improving health literacy for lower-educated individuals.

